# Deciphering the mitochondria–gut microbiota axis in irritable bowel syndrome: pathogenic insights and therapeutic frontiers

**DOI:** 10.1186/s12967-026-07894-9

**Published:** 2026-03-02

**Authors:** Lifang Zeng, Wenpeng Zhou

**Affiliations:** 1 Department of Gastroenterology, The First Affiliated Hospital of Jinan University, Jinan University, Guangzhou, 510630 China; 2 Department of Gastroenterology and Hepatology, The First Affiliated Hospital, Sun Yat-sen University, Guangzhou, 510080 P.R. China; 3 Department of Gastroenterology, Guangdong Provincial Key Laboratory of Major Obstetric Diseases, Guangdong Provincial Clinical Research Center for Obstetrics and Gynecology, The Third Affiliated Hospital, Guangzhou Medical University, Guangzhou, 510150 China

**Keywords:** Mitochondria, Gut microbiota, Irritable bowel syndrome, Pathogenesis, Therapy

## Abstract

**Background:**

Irritable bowel syndrome (IBS) is a chronic gastrointestinal disorder involving multiple pathogenic mechanisms. Gut microbiota dysbiosis and mitochondrial dysfunction are key drivers of IBS, as they weaken the intestinal barrier, promote inflammation, and alter metabolism. However, the underlying mechanisms of crosstalk between these factors remain largely understudied, which hinders the clinical translation of targeted therapeutic strategies.

**Main body:**

Drawing on recent literature, this review clarifies the intricate interplay between mitochondrial health and gut microbiota balance in IBS. We summarize the core pathophysiological mechanisms of IBS including neuroendocrine, immune-inflammatory, and gastrointestinal motility-related pathways. We further elaborate on the individual roles of mitochondrial dysfunction and gut microbiota dysbiosis in IBS pathogenesis, discussing the bidirectional crosstalk between mitochondria and the gut microbiota, and the regulatory role of diet in this axis. Current therapeutic approaches targeting IBS, such as Western medications; probiotics; fecal microbiota transplantation; low-fermentable oligosaccharide, disaccharide, monosaccharide, and polyol diet; traditional Chinese medicine; and mitochondria-targeted interventions, are also reviewed. Building on this, the review summarizes the advantages and limitations of existing research on the mechanisms and therapies for IBS and identifies the challenges and directions for future basic and clinical research.

**Conclusions:**

The interplay between mitochondrial dysfunction and gut microbiota dysbiosis is a critical research focus for understanding IBS pathophysiology. Elucidating the underlying mechanisms of their crosstalk provides a foundation for future research and facilitates the development of innovative therapeutic strategies targeting mitochondrial health and gut microbiota balance. This multifaceted approach holds promise for improving IBS management and enhancing the quality of life of affected patients.

## Introduction

**Fig. 1:**
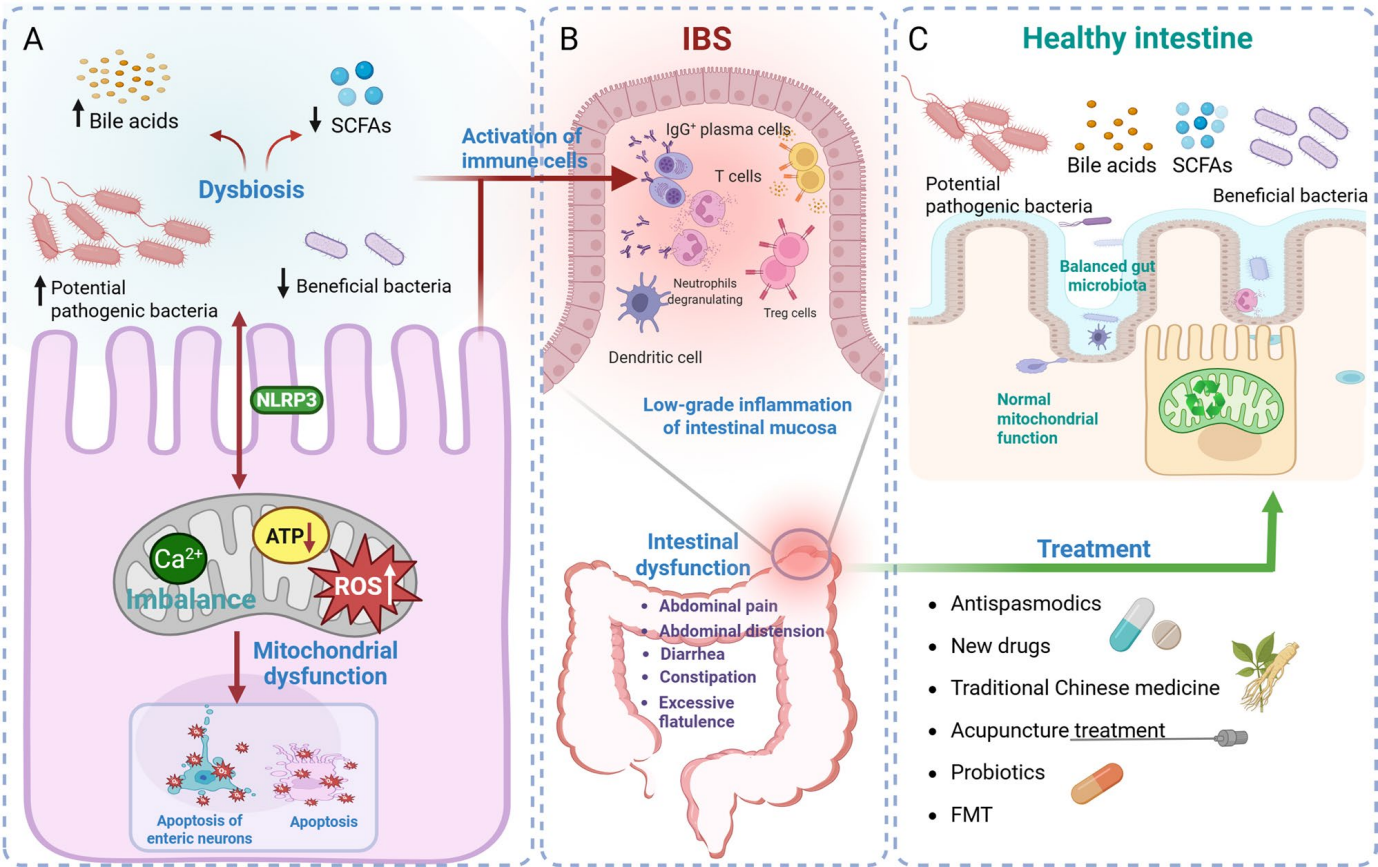
Mitochondria–gut microbiota interaction in Irritable bowel syndrome (IBS) pathogenesis and relevant treatment methods. **A** Gut microbiota dysbiosis increases the abundance of potential pathogenic bacteria and decreases that of beneficial ones, increases the levels of bile acids, and reduces short-chain fatty acid (SCFA) levels. This activates pathways, resulting in intracellular Ca²⁺ imbalance, reduced adenosine triphosphate (ATP) production, increased reactive oxygen species (ROS) production, mitochondrial dysfunction, metabolic abnormalities, and ultimately, enteric neuron apoptosis, consequently disrupting intestinal function. Conversely, mitochondrial dysfunction can also trigger gut microbiota dysbiosis. **B** Dysbiosis and mitochondrial dysfunction jointly activate immune cells such as T cells and neutrophils, leading to low-grade intestinal mucosal inflammation, intestinal dysfunction, and IBS symptoms such as abdominal pain, diarrhea, and constipation. **C** Intervention with one or more novel drugs, traditional Chinese medicine (TCM), acupuncture, probiotics, and fecal microbiota transplantation (FMT) improves the gut microbiota and mitochondrial function

Irritable bowel syndrome (IBS) is a prevalent functional gastrointestinal disorder that is predominantly characterized by abdominal pain, bloating, and perturbations in bowel habits [1, 2]. Although the exact etiology of IBS remains unclear, mitochondrial dysfunction and gut microbiota dysbiosis may play crucial roles in the pathogenesis of this disorder (Fig. 1A). The gut microbiota affects mitochondrial function through metabolites, leading to mitochondrial dysfunction, which consequently interferes with the host’s metabolic and immune status [3] and promotes the development of IBS. The ability to immunologically monitor and regulate the gut microbiota declines during mitochondrial dysfunction, which may trigger alterations in the intestinal environment, promote microbiota dysbiosis, and induce the onset of IBS symptoms [4–6].

Interventions targeting the gut microbiota have opened new avenues for the improvement of IBS symptoms. Potential strategies such as probiotics and nutritional interventions [7], treatments targeting mitochondria, and traditional Chinese medicine (TCM) not only improve the composition of the gut microbiota [8] but may also alleviate IBS symptoms by improving mitochondrial function [9] (Fig. 1C). Restoration of the gut microbiota is intricately associated with the improvement of mitochondrial function, thereby providing novel insights and directions for IBS treatment [10].

In conclusion, the interplay between mitochondrial dysfunction and gut microbiota dysbiosis is a promising area of research for understanding IBS. Elucidating the underlying mechanisms can enhance our understanding of IBS pathophysiology and facilitate the development of innovative therapeutic strategies that target mitochondrial health and gut microbiota balance. This multifaceted approach can ultimately lead to better management of IBS and an elevated quality of life in patients with this challenging condition.

**Fig. 2:**
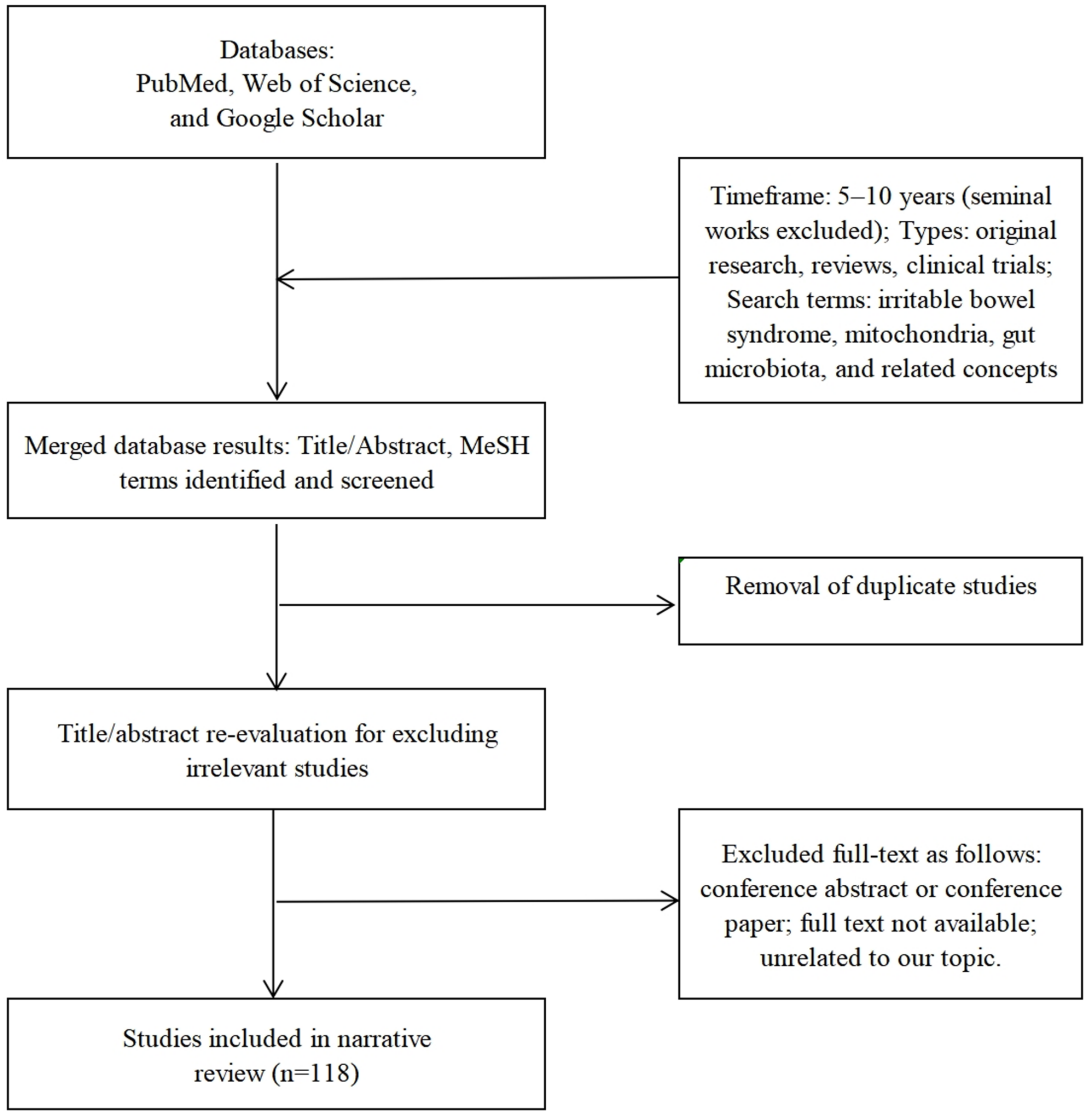
Literature search and screening flowchart

This review explored the crosstalk between the gut microbiota and mitochondria in IBS. We searched for papers in multiple databases, including PubMed, Web of Science, and Google Scholar. We applied no language restrictions. The search covered all studies, including original research, reviews, and clinical trials ranging from in vitro and in vivo to human studies. However, we primarily focused on articles published within the past 5–10 years (except for seminal works directly relevant to the topic of research). The following search terms were employed: “irritable bowel syndrome,” “mitochondria,” “gut microbiota,” and their related concepts. The basic search strategy was: ((((((irritable bowel syndrome) OR (irritable bowel syndrome [MeSH Terms])) OR (irritable bowel syndrome [Title/Abstract])) OR (IBS)) OR (IBS [Title/Abstract])) OR (((mitochondria) OR (mitochondria [MeSH Terms])) OR (mitochondria [Title/Abstract]))) OR (((gut microbiota) OR (gut microbiota [MeSH Terms])) OR (gut microbiota [Title/Abstract])); (((((irritable bowel syndrome) OR (irritable bowel syndrome [MeSH Terms])) OR (irritable bowel syndrome [Title/Abstract])) OR (IBS)) OR (IBS [Title/Abstract])) AND (((mitochondria) OR (mitochondria [MeSH Terms])) OR (mitochondria [Title/Abstract])); (((((irritable bowel syndrome) OR (irritable bowel syndrome [MeSH Terms])) OR (irritable bowel syndrome [Title/Abstract])) OR (IBS)) OR (IBS [Title/Abstract])) AND (((gut microbiota) OR (gut microbiota [MeSH Terms])) OR (gut microbiota [Title/Abstract])); (((mitochondria) OR (mitochondria [MeSH Terms])) OR (mitochondria [Title/Abstract])) AND (((gut microbiota) OR (gut microbiota [MeSH Terms])) OR (gut microbiota [Title/Abstract])); ((((((irritable bowel syndrome) OR (irritable bowel syndrome [MeSH Terms])) OR (irritable bowel syndrome [Title/Abstract])) OR (IBS)) OR (IBS [Title/Abstract])) AND (((mitochondria) OR (mitochondria [MeSH Terms])) OR (mitochondria [Title/Abstract]))) AND (((gut microbiota) OR (gut microbiota [MeSH Terms])) OR (gut microbiota [Title/Abstract])). The exclusion criteria were as follows: conference abstracts or conference papers; full text not available; unrelated to our topic; and duplicated studies. Finally, 118 articles were included in the review to comprehensively present the latest research on this topic (Fig. 2).

## Pathophysiological mechanisms

### Core pathophysiological mechanisms related to IBS

#### Neuroendocrine mechanisms

IBS is affected by neuroendocrine mechanisms, particularly through the brain–gut axis. This axis represents complex communication between the gastrointestinal tract and the central nervous system, in which neuroendocrine factors play a vital role. Some gut bacteria and their metabolites can synthesize neurotransmitters, consequently triggering the enteric nervous system and influencing central nervous system function. Thus, they facilitate the persistence of IBS symptoms, namely abdominal pain, abdominal distention, alterations in bowel habits, anxiety, and depression states [11–13].

Stress leads to changes in corticotropin-releasing hormone levels. This can affect intestinal motility and increase visceral hypersensitivity, which is a typical feature of IBS [14]. Additionally, dysregulation of the hypothalamus–pituitary–adrenal axis is associated with abnormal cortisol secretion. This affects the gut microbiota and subsequently contributes to the pathophysiology of IBS, thereby exacerbating its symptoms [15, 16]. Furthermore, epigenetic mechanisms, including DNA methylation and histone modifications, are potential regulators of gene expression in response to environmental stressors. These mechanisms highlight the role of neuroendocrine factors in the emergence and continuation of IBS symptoms [17]. In a recent study involving 80 patients with IBS and 80 healthy individuals [18], patients with IBS showed different bacterial genera from healthy patients. These differences were associated with higher levels of neuroendocrine substances such as adrenocorticotropic hormone, cortisol, and 5-hydroxytryptamine [18].

The brain–gut axis plays a crucial role in the occurrence of IBS. Moreover, the interaction between the brain and the gut is bidirectional. This provides a foundation for the development of therapies that target the neuroendocrine components of IBS and may lead to more effective treatment strategies. The role of the brain–gut axis in the pathogenesis of IBS is complex, and current research has not fully clarified all aspects. Therefore, more in vitro and in vivo experiments are required to determine the specific mechanism underlying this process and provide new directions for IBS treatment.

#### Immune response and inflammation

Immune response is a critical aspect of IBS pathogenesis, with emerging evidence suggesting that low-grade inflammation has vital implications (Fig. 1B). Patients with IBS often exhibit signs of immune dysregulation characterized by elevated levels of inflammatory cytokines and altered gut permeability [19, 20]. Immune dysregulation may result in chronic gut inflammation and manifest as symptoms such as abdominal pain and perturbed bowel habits. Activation and degranulation of immune cells, such as mast cells, in the gut mucosa are major causes of abdominal pain in IBS [21–23]. Nozu et al. [24] revealed that tranilast inhibits the release of interleukin-1β (IL-1β) from NLRP3 inflammasome activation to alleviate IBS symptoms in rat models.

The crosstalk between mitochondria and gut microbiota exerts a pivotal effect on the modulation of immune responses. Mitochondrial dysfunction further exacerbates immune-inflammatory responses by regulating gut microbiota imbalance. Both mitochondria and the gut microbiota are indispensable components of the host immune system. They affect inflammation and immune regulation via multiple mechanisms. Mitochondrial dysfunction can impair immune responses, resulting in inflammation and autoimmune diseases [25]. For example, reactive oxygen species (ROS) produced by mitochondria can act as signaling molecules to activate the nuclear factor kappa B pathway, which is a key regulator of inflammation [3]. Moreover, the role of the gut microbiota in the regulation of immune responses cannot be overlooked, because dysbiosis, also known as an imbalance within the gut microbiota, is associated with IBS. Specific microbial profiles, including increased proinflammatory bacteria and decreased numbers of beneficial species, have been observed in patients with IBS [26]. A review that analyzed several studies on patients with IBS indicated that the gut microbiota can induce inflammation by activating the immune system, which leads to IBS [27]. These findings suggest that targeting the immune response and restoring microbial balance may provide therapeutic benefits for individuals with IBS. However, additional investigations are necessary to elucidate the exact mechanisms through which inflammation and immune responses lead to IBS symptoms.

In summary, the combined roles of mitochondria and the gut microbiota in immune responses highlight their significance in health and disease. Their interactions affect inflammation, immune regulation, and overall homeostasis, thereby providing potential avenues for therapeutic interventions in various inflammatory and metabolic diseases. Further research elucidating these complex interactions could facilitate the development of strategies to enhance immune function and mitigate disease progression, including that of IBS.

#### Gastrointestinal motility dysfunction

Gastrointestinal motility dysfunction is a core feature of IBS. It manifests as either constipation or diarrhea, depending on the subtype of the condition. Abnormalities in gut motility can arise from various factors, including altered neural signaling, hormonal imbalances, and changes in gut microbiota composition [28]. For instance, alterations in serotonin signaling, particularly through the serotonin transporter in both mice and humans, may disrupt normal gut motility patterns, and may eventually lead to the onset of IBS symptoms [29]. Li et al. [30] attributed accelerated colonic motility in an IBS-diarrhea (IBS-D) rat model to the activation of enteric glial cells in the myenteric plexus. Similarly, a study employing IBS mouse models showed considerably higher frequency and amplitude of spontaneous contractions in the proximal colon segments [31]. These results indicate that gastrointestinal motility dysfunction is a major cause of IBS symptoms.

The link between the gut microbiota and gastrointestinal motility is of utmost significance, as microbial metabolites such as short-chain fatty acids (SCFAs) can affect peristalsis and gut transit time in different animal models [32]. Studies have further emphasized the contribution of gut–brain communication in the regulation of motility, which suggests that microbiota and psychological factors exacerbate dysmotility in patients with IBS [33]. Therefore, more histological experiments or observational studies involving animals and humans should be conducted to elucidate the mechanisms underlying these motility dysfunctions. This research scope is critical for the development of targeted therapies to restore normal gut function and alleviate IBS symptoms.

In summary, the neuroendocrine mechanisms between the brain and gut, immune responses and inflammation in the gut, and gastrointestinal motility dysfunction are the main mechanisms underlying IBS. Although many studies have evaluated these pathophysiological mechanisms, the exact molecules and related signaling pathways warrant further clarification using in vivo and in vitro experiments. Furthermore, other mechanisms need to be explored.

### Roles and mechanisms of mitochondrial dysfunction in IBS

#### Mitochondrial function and gut health

Mitochondria are essential organelles responsible for the production of adenosine triphosphate (ATP), an intracellular energy carrier, via oxidative phosphorylation. Gut health is closely linked to mitochondrial function, as these organelles preserve the integrity of the intestinal barrier and regulate inflammatory responses. A study using an IBS mouse model revealed that mitochondrial dysfunction impairs ATP production in mouse intestinal stem cells, which leads to reduced self-renewal and differentiation of these cells. This impairs intestinal epithelial regeneration and gut homeostasis [34]. Rodríguez-Colman et al. [35] demonstrated that intestinal stem cells (also called Lgr5^+^ crypt base columnar cells) exhibit high mitochondrial activity in the mouse small intestine, which can maintain stem cell function and further contribute to intestinal homeostasis. These studies demonstrate that mitochondrial function is crucial for intestinal homeostasis, and that any factors that weaken mitochondrial function are harmful to gut health.

#### Mitochondrial dysfunction and IBS

In IBS, mitochondrial dysfunction is associated with altered energy metabolism that can lead to augmented intestinal transmissibility and abnormal immune responses. A study using an IBS rat model indicated that compromised mitochondrial bioenergetics, resulting in decreased ATP production and enhanced ROS generation, contribute to oxidative stress and irritation in the gut [36]. Furthermore, a study using public sequencing data and in vitro experiments showed that excessive ROS production resulting from mitochondrial dysfunction can cause cell oxidative damage [37]. These studies demonstrate that oxidative stress can disrupt gut epithelial integrity and promote inflammatory pathways that result in the onset IBS symptoms. Moreover, dietary interventions aimed at improving mitochondrial function, such as ketogenic diets, have demonstrated the potential to alleviate IBS symptoms by enhancing mitochondrial biogenesis and reducing gut inflammation [36]. In a previous study involving both in vitro and mouse experiments [38], mitochondrial damage in enteric glial cells impaired neuronal function and increased gut inflammation. Therefore, mitochondrial health is crucial in the management of IBS.

Mitochondrial dysfunction correlates with IBS symptoms and pathogenesis. Alterations in mitochondrial dynamics in male rat models of IBS, including changes in mitochondrial morphology and reduced bioenergetic capacity, correlate with the severity of IBS symptoms [39]. Moreover, the mitochondrial DNA copy number is associated with disease severity in male patients with IBS. Gender-related differences were observed in the expression of mitochondrial ATP 6/8 genes in patients with diarrhea-predominant IBS [40, 41]. Specifically, mitochondrial ATP 6/8 genes are more frequently expressed in patients with IBS characterized by diarrhea [40]. The duplicate number of mitochondrial DNA also correlates with male sex and drinking habits [41]. The aforementioned studies confirm that mitochondrial dysfunction (such as reduced ATP production, excessive ROS generation, and dynamic changes) is closely associated with the severity of IBS symptoms. Thus, mitochondrial DNA copy number and ATP 6/8 gene expression have the potential to serve as diagnostic markers. The advantages of these studies lie in revealing the pathogenesis of IBS from the perspectives of energy metabolism and oxidative stress. However, the aforementioned studies have some limitations. First, most clinical studies on the subject are cross-sectional analyses that lack longitudinal data to verify the causal relationship between mitochondrial dysfunction and IBS. Moreover, existing studies have not clarified the characteristics of differences in mitochondrial function among IBS subtypes (e.g., constipation-predominant IBS [IBS-C] and IBS-D). Therefore, prospective investigations and intervention trials are required to further verify the clinical value of mitochondria-targeted strategies.

### Pathogenic role of gut microbiota dysbiosis in IBS

#### Functions of normal gut microbiota

The gut microbiota is crucial for sustaining gastrointestinal health and overall well-being. It participates in diverse physiological functions, such as digestion, metabolism, and immune modulation. A multi-center study of Asian patients with IBS indicated that balanced gut microbiota maintain gut barrier integrity [42]. Furthermore, crosstalk between the gut microbiota and the host immune system promotes immune tolerance and prevents pathogen colonization [43]. An equilibrated gut microbiota is necessary to prevent dysbiosis, which can lead to various gastrointestinal dysfunctions, including IBS [44]. The gut microbiota can produce neurotransmitters, neurotrophic factors, and metabolites that maintain the gut barrier, occlude junction integrity, and ultimately regulate the central and intestinal nervous systems [45, 46]. In summary, the gut microbiota is pivotal to preserving human health.

#### Features of the gut microbiota in patients with IBS

Patients with IBS frequently exhibit distinct modifications in gut microbiota composition relative to healthy controls. Studies have shown diminished microbial diversity and specific shifts in microbial populations. Pecyna et al. [47] analyzed stool samples from 121 patients with IBS (70 women and 51 men) and 70 non-IBS individuals (40 women and 30 men) using 16 S ribosomal ribonucleic acid (16 S rRNA) gene sequencing. The populations of potentially pathogenic microbes, including *Anaerovorax* spp., *Clostridiales*, *Coriobacteriaceae*, *Mogibacteriaceae*, and *Synergistaceae*, were increased in patients with IBS, whereas those of beneficial taxa, such as *Shuttleworthia* spp., were decreased [47]. Pecyna et al. [47] further analyzed gender-related differences in gut microbiota composition among patients with IBS, revealing that female patients exhibit higher abundances of *Clostridiales* and *Synergistaceae*. Furthermore, differences in the gut microbiota were not observed among different IBS types (including IBS-C and IBS-D) [47]. However, this was a single-center comparative study, and the functional implications of microbiota differences were not clarified, which consequently affected its application. A study based on sequencing data from the American Gut Project confirmed prior findings, demonstrating that beneficial bacteria (*Faecalibacterium* and *Bifidobacterium*) and pathogenic bacteria (*Escherichia/Shigella*) exhibit opposing abundance patterns in patients with IBS [48]. In addition, Su et al. [48] reported diverse gut microbiota profiles among different IBS subtypes. For instance, subtype-specific signatures include *Ruminococcus gnavus* enrichment in IBS-D and *Oscillospira spp.* in IBS-C. Moreover, female patients with IBS were more prone to gut dysbiosis. Although data from 942 patients with IBS were used in the study by Su et al., the results from this observational cross-sectional analysis were influenced by potential confounders and the use of questionnaires. In another study, some genera, including *Muricauda* (phylum Bacteroidetes), *Pelagerythrobacter*, and *Rickettsia* (phylum Proteobacteria), were highly abundant in patients with IBS [49]. Furthermore, the abundance of microbes varied among different IBS subtypes. In particular, *Peptoniphilus*, *Haemophilus*, and *Roseburia* were enriched in IBS-D, whereas *Anaerofilum* was enriched in IBS-C [49]. Liu et al. [50] revealed the abundance of the phylum Actinobacteria and the genus *Flavonifractor* as risk factors for IBS, and that of the genus *Eisenbergiella* as a protective factor. However, the aforementioned studies used publicly available data. Research using public data has some limitations, such as lack of sample uniformity across different regions and the lack of corresponding clinical characteristics.

Findings from analyses of the human microbiome suggest that the gut microbiota plays a pivotal role in the pathogenesis of IBS. In summary, existing studies consistently indicate that patients with IBS exhibit gut microbiota dysbiosis characterized by a decreased abundance of beneficial bacteria and increased abundance of potentially pathogenic bacteria. Additionally, this imbalance may be associated with IBS subtypes and sex. However, current research has obvious limitations. First, most studies have small sample sizes, some use single-center designs, and sequencing methods are mainly based on 16 S rRNA. Consequently, complete elucidation of functional differences of the microbiota remains difficult. Second, confounding factors such as geographical differences and dietary interventions are not fully controlled, leading to heterogeneity among different research results. Specifically, 16 S rRNA sequencing cannot achieve accurate species-level identification. Third, the insufficient application of shotgun metagenomics is one of the major limitations in this research field; it has led to the lack of sufficient whole-genome data to support the prediction of gut microbiota functions in IBS. Therefore, future multi-center, large-sample cohort studies combining metagenomic sequencing with clinical characteristic analysis are required to identify IBS-specific microbiota biomarkers and their pathogenic mechanisms. Specifically, future studies should adopt multi-omics techniques integrating 16 S rRNA sequencing, shotgun metagenomics, and metabolomics to comprehensively clarify the structural and functional characteristics of the gut microbiota in IBS.

#### Impact of microbiota dysbiosis on IBS

##### Impact of microbiota dysbiosis on the intestinal barrier

Dysbiosis is an imbalance within the gut microbiota, marked by changes in microbial diversity, an excess of harmful bacteria, and decreased beneficial bacteria [51]. Detection methods for dysbiosis typically involve sequencing methods such as 16 S rRNA gene sequencing, thereby allowing comprehensive profiling of microbial communities [47]. Additionally, metabolomic analyses can complement assessments of the microbiota by identifying metabolic byproducts indicative of dysbiosis, such as altered SCFA levels [52]. These methods elucidate the microbial landscape and its functional impact on IBS.

Gut dysbiosis can significantly impair intestinal barrier function, leading to an uptick in permeability, commonly referred to as “leaky gut.” This disruption enables bacteria and their metabolites to translocate into the bloodstream, thereby initiating systemic inflammation and worsening disease symptoms [53]. Beneficial gut bacteria ferment dietary fibers to generate SCFAs, which possess anti-inflammatory properties and contribute to the maintenance of gut barrier function [42]. Dysbiosis is associated with the depletion of SCFA-producing bacteria that are vital for maintaining the integrity of intestinal epithelial cells and sustaining gut homeostasis [48]. Consequently, a compromised intestinal barrier can contribute to the pathogenesis of IBS by triggering a cycle of inflammation and dysbiosis [44]. Dysbiosis often leads to reduced SCFA production, subsequently exacerbating IBS symptoms by impairing intestinal motility and increasing sensitivity [53]. Hence, gut microbiota metabolites, particularly SCFAs, are essential for modulating gut health and mitigating IBS symptoms.

##### Impact of microbiota dysbiosis on the immune system

Dysbiosis significantly affects the immune system and alters both local and systemic immune reactions in humans. Analysis of fecal samples from 30 patients with IBS-D and 30 healthy controls revealed that dysbiosis may cause dysregulation of proinflammatory and anti-inflammatory cytokines, thereby contributing to persistent gut inflammation [51]. For instance, patients with IBS often exhibit increased levels of inflammatory markers, including interleukin-6 (IL-6) and tumor necrosis factor-α (TNF-α), which correlate with dysbiosis [53]. Moreover, the gut microbiota shape the immune landscape; they facilitate the differentiation of T helper cells and the production of immunoglobulins, consequently affecting the overall immune response to gut pathogens [53]. Immune response is an essential element in the pathogenesis of IBS, especially in relation to dysbiosis. Immune activation can cause visceral hypersensitivity, a characteristic symptom of IBS, by intensifying pain perception and gut motility [54]. Additionally, autoantibodies directed against the enteric nervous system have been observed in patients with IBS, indicating a significant role of the autoimmune response in this disease [55]. Immune dysregulation may exacerbate gut inflammation and lead to chronic IBS symptoms [56]. Furthermore, metabolites, including bile acids and indole derivatives, are associated with gut signaling pathways that regulate motility and inflammation. This demonstrates their potential therapeutic role in IBS management [57].

The interplay between dysbiosis and IBS pathogenesis is complex, involving multiple factors. Dysbiosis can alter intestinal barrier function, immune dysregulation, and metabolic disturbances, all of which contribute to the occurrence and intensification of IBS symptoms [43]. Thus, clarifying the specific mechanisms is critical for developing targeted therapies aimed at restoring gut microbiota homeostasis and enhancing the prognosis of patients with IBS.

### Mitochondria and the gut microbiota

Preclinical and clinical studies revealing the interaction between the mitochondria–gut microbiota axis and IBS have distinct research approaches. In particular, they differ substantially in terms of evidence sources. Preclinical studies mostly rely on cell models (such as intestinal epithelial and enteric neural cells) and animal (such as IBS rats and mice). Thus, these study types can accurately control variables and explore molecular mechanisms more comprehensively. In contrast, clinical studies are mainly based on human cohorts and randomized controlled trials (RCTs), directly reflecting the pathophysiological state in real clinical scenarios. Preclinical and clinical studies also have differing research objects. Preclinical research objects have high homogeneity, thereby facilitating mechanism verification. In contrast, clinical research objects have individual differences (such as age, underlying diseases, and living habits); therefore, the results are more representative but highly heterogeneous. From the perspective of evidence strength, preclinical studies can establish causal relationships through intervention experiments (such as gene editing and microbiota transplantation), with strong evidence strength. However, species differences may lead to difficulties in the direct translation of results. Although RCTs in clinical studies have relatively high evidence strength, ethical constraints make it difficult to explore mechanisms using invasive approaches. In terms of application scope, preclinical research results provide targets and theoretical bases for clinical studies. Thus, they are suitable for mechanism verification in the early stage of drug development. Clinical research results directly guide clinical practice and are consequently suitable for treatment plan optimization and guideline formulation. In summary, the two study types have complementary advantages, and a closed-loop research framework of ‘preclinical mechanism verification–clinical translation and application’ is required to fully clarify the role of the mitochondria–gut microbiota axis in IBS.

#### Crosstalk between mitochondria and the gut microbiota

Mitochondria can regulate the makeup and functionality of the gut microbiota and function as an energy source for cells. They also form an integral part of the maintenance of cellular homeostasis, including the gut environment. Dysfunction of mitochondrial metabolism can alter ROS production, which affects the diversity and quantity of the gut microbiota. For instance, changes in mitochondrial genes can significantly affect gut microbiota composition, thereby suggesting a direct link between mitochondrial function and microbiota dynamics [58]. Disruptions in this relationship may result in a range of gastrointestinal disorders, including colorectal cancer and inflammatory bowel disease (IBD) [58]. Additionally, mitochondrial health affects the generation of metabolites, including SCFAs, by the interacting microbiota, thereby establishing a feedback loop that underscores the importance of mitochondrial integrity in sustaining a balanced gut microbiota [59]. These studies demonstrate that mitochondrial dysfunction of intestinal cells may cause gut microbiota dysbiosis.

The gut microbiota can also significantly affect mitochondrial function. Emerging evidence suggests that microbial metabolites such as SCFAs modulate mitochondrial bioenergetics, dynamics, and stress responses. Table 1 highlights the key gut microbiota-derived metabolites and their regulatory effects on mitochondrial processes, which are central to the crosstalk between the two systems. These metabolites boost mitochondrial respiration and ATP generation, consequently affecting the cellular energy metabolism [60]. SCFAs, such as butyrate, provide energy to colonocytes and enhance mitochondrial biogenesis and oxidative phosphorylation [61]. Impaired mitochondrial function across multiple tissues has been linked to perturbations in gut microbiota composition, thereby exacerbating oxidative stress and mitochondrial inflammation [62]. Additionally, bile acids produced by gut bacteria regulate mitochondrial function in liver cells, thereby affecting lipid metabolism and energy homeostasis [62]. For instance, dysbiosis can result in decreased SCFA production, thereby potentially impairing mitochondrial function and contributing to metabolic disorders, including type 2 diabetes and obesity [63]. This interplay implies that sustaining a healthy gut microbiota is key to optimal mitochondrial performance and overall health. Although the causal relationship between the two remains incompletely elucidated, their bidirectional crosstalk via multiple signaling pathways is crucial for maintaining optimal health.

**Table 1:** Key metabolites and their effects on mitochondrial processes

Metabolite	Source	Effects on mitochondrial processes	References (Corresponding lines in the manuscript)
Short-chain fatty acids (SCFAs, e.g., butyrate)	Fermentation of dietary fibers by beneficial gut bacteria (e.g., *Faecalibacterium*, *Bifidobacterium*)	(1) Provide direct energy substrates for colonocytes; (2) Enhance mitochondrial biogenesis; (3) Promote oxidative phosphorylation to increase ATP production; (4) Upregulate the expression of mitochondrial biogenesis-related genes (e.g., *PGC-1α*)	Explicitly correspond to references [32, 42, 59, 61] and [64] in the manuscript
Bile acids	Synthesized by gut bacteria	(1) Regulate mitochondrial function in liver cells; (2) Modulate mitochondrial processes related to lipid metabolism; (3) Maintain energy homeostasis through mitochondrial regulation	Explicitly corresponds to reference [62] in the manuscript
Lipopolysaccharides	Metabolites of pathogenic bacteria during gut microbiota dysbiosis	(1) Induce the production of mitochondrial reactive oxygen species; (2) Exacerbate mitochondrial oxidative stress; (3) Disrupt the normal mitochondrial respiratory chain function indirectly by regulating proinflammatory cytokines (e.g., TNF-α and IL-6)	Derived from references [53] and [58] in the manuscript
Indole derivatives	Metabolites of the gut microbiota	(1) Regulate mitochondrial stress response; (2) Participate in gut signaling pathways associated with the regulation of mitochondrial-mediated inflammation; (3) Maintain mitochondrial functional stability under pathological conditions	Extended and supplemented based on reference [57] in the manuscript

The interaction between mitochondria and gut microbiota-derived metabolites is complicated by diet—nutritional components directly affect mitochondrial function and microbial composition, forming a tripartite regulatory network. For example, diets containing soluble and insoluble fiber promote the proliferation of beneficial gut bacteria that generate SCFAs (e.g., *Faecalibacterium* and *Bifidobacterium*); these SCFAs not only serve as direct energy substrates for mitochondrial oxidative phosphorylation but also upregulate the expression of genes related to mitochondrial biogenesis (e.g., *PGC-1α*) [42, 64]. Another study revealed that a ketogenic diet could restore mitochondrial function to reduce inflammation and oxidative stress in IBS rats compared with a standard diet [36]. This also suggests that diet first affects mitochondrial function, which may subsequently influence the gut microbiota. Thus, dietary interventions may serve as a therapeutic strategy for regulating the mitochondria–gut microbiota axis and enhancing health outcomes. However, more research should focus on the regulatory mechanisms between diet and the mitochondria–gut microbiota axis in IBS.

Although the causal association between mitochondria and the gut microbiota has not been fully elucidated, their interaction through diverse signaling pathways is crucial for human health. Understanding these interactions provides insight into potential therapeutic targets for disease and underscores the importance of dietary interventions in the modulation of these pathways. In summary, more research using standardized methodologies and experimental models is required to clarify whether there is a clear causal relationship between mitochondria and the gut microbiota, while further exploring the regulatory role of diet in this axis.

Dietary heterogeneity in existing studies poses challenges to the interpretation of the microbiota–mitochondria relationship. Differences in fiber intake levels, macronutrient ratios (e.g., carbohydrate–fat balance), and dietary patterns (e.g., Western vs. Mediterranean diets) may mask the true association between microbial metabolites and mitochondrial function [12, 42]. For example, inconsistent fiber intake among study populations can lead to variable SCFA production, resulting in conflicting findings regarding mitochondrial bioenergetic changes. Future studies should adopt standardized dietary interventions or stratified analysis based on dietary characteristics to reduce these confounding effects.

#### Maintenance of gut homeostasis by mitochondria and the gut microbiota

The connection between the gut microbiota and mitochondria is vital for preserving intestinal homeostasis. Mitochondrial dysfunction may induce an imbalance in the gut microbiota. This exacerbates mitochondrial damage, thereby creating a vicious cycle that leads to the development of gastrointestinal diseases [59]. For instance, conditions that disrupt mitochondrial function, such as oxidative stress, can shift the balance to favor the expansion of pathogenic bacteria over beneficial ones. Dysbiosis increases intestinal permeability and inflammation, thereby further compromising mitochondrial health [65]. Thus, collaboration between these two systems is vital for the preservation of gut integrity and function.

The link between the gut microbiota and mitochondria also modulates immune responses. Dysbiosis has been linked to chronic inflammation and immune dysregulation, which can affect mitochondrial function and vice versa [65]. For instance, microbial metabolites can alter the stimulation of immune cells and the production of inflammatory cytokines, thereby affecting mitochondrial dynamics and function [58]. This crosstalk is particularly pertinent in autoimmune diseases and metabolic disorders in which manifestations of both mitochondrial impairment and gut microbiota alterations are observed. Thus, understanding these interactions may offer new therapeutic approaches for the management of immune-related conditions.

#### Effects of microbiota–mitochondria interactions on IBS

The interplay between mitochondria and the gut microbiota has ramifications for multiple signaling pathways. Bioinformatic analysis of gut microbiota dysbiosis in mice revealed that microbial metabolites can modulate key signaling pathways linked to inflammation, metabolism, and cell survival, including the NLRP3 inflammasome and the mitogen-activated protein kinase signaling pathway [66]. For example, SCFAs can activate G-protein-coupled receptors in host cells, consequently triggering pathways including the adenosine monophosphate–activated protein kinase pathway, which is essential for maintaining energy homeostasis and ensuring proper mitochondrial function [3]. These pathways are indispensable in the maintenance of cellular homeostasis and stress responses. Their dysregulation due to altered gut microbiota composition can cause mitochondrial dysfunction and participate in the pathogenesis of diseases such as metabolic syndrome and IBD [61]. This indicates that metabolites from the gut microbiota not only directly affect mitochondrial function but also engage in complex signaling cascades that regulate metabolic processes in the host. Targeting these pathways through dietary interventions or microbiota modulation may offer potential disease prevention and treatment strategies. Dysbiosis has been linked with the progression of IBS, potentially via mechanisms involving mitochondrial dysfunction [62]. Alterations in gut microbiota composition can increase the formation of gas and metabolites that may exacerbate mitochondrial stress and contribute to the symptoms of IBS. Furthermore, the inflammatory responses triggered by dysbiosis can compromise mitochondrial function, subsequently creating a cycle of dysfunction that perpetuates the condition [60]. Gut microbiota dysbiosis can exacerbate mitochondrial stress by increasing gas and metabolite production, thereby worsening IBS symptoms. Meanwhile, the inflammatory response triggered by dysbiosis impairs mitochondrial function, forming a vicious cycle that perpetuates the condition. Future studies aiming to reveal these interactions may provide insights into novel therapeutic approaches for IBS. Additionally, appropriate therapeutic approaches that restore gut microbiota balance and mitochondrial health must be proposed. Based on the above mechanisms, targeted intervention of mitochondrial function and intestinal flora balance has become an important direction for the treatment of IBS.

## Therapeutic approaches and future directions

### Current status and limitations of clinical treatments for IBS

IBS, a highly prevalent functional gastrointestinal disorder, substantially compromises patient quality of life. Recent advancements in the clinical treatment of IBS have focused on pharmacological and non-pharmacological strategies to address the multifactorial nature of this disorder. IBS management has evolved to incorporate various treatment modalities, including psychological therapies, dietary modifications, and pharmacological interventions, thereby securing a more holistic approach to patient care. Studies have suggested that a multidisciplinary strategy that combines these modalities can yield better outcomes for patients with IBS, while emphasizing the importance of personalized therapeutic regimens tailored to individual patient needs and specific symptom profiles [67, 68].

Clinical trials investigating the efficacy of microbiota-targeted interventions such as fecal microbiota transplantation (FMT) and probiotics against IBS share common methodological flaws that undermine their scientific rigor and clinical relevance. First, small sample sizes (mostly ≤ 100 cases) and single-center designs limit statistical power and external validity, leading to inconsistent results across studies. Second, the lack of stratified analysis by IBS subtypes (IBS-D/IBS-C/mixed-diarrhea-and-constipation IBS [IBS-M]) and baseline characteristics (e.g., gut microbiota composition and mitochondrial function) ignores the heterogeneous nature of IBS, thereby masking subgroup differences in treatment response. Third, short follow-up periods (FMT: <12 months; probiotics: <6 months) fail to capture long-term efficacy and potential safety risks (e.g., FMT-related infection and probiotic-induced dysbiosis). Fourth, non-standardized efficacy evaluation systems (e.g., variable symptom scoring scales and inconsistent gut barrier function detection indicators) hinder cross-study comparisons. These limitations have significant implications for clinical decision-making. In particular, current evidence remains inadequate to endorse the routine application of FMT or probiotics as first-line treatments for IBS. Instead, they should be considered as exploratory interventions in clinical trials or refractory cases. Future trials must address these flaws by adopting multi-center large-sample designs, stratified randomization, standardized outcome measures, and long-term follow-up to enhance the reliability and translatability of findings.

### Western treatment progress of IBS

The main treatment strategies for IBS (summarized in Table 2) have undergone significant advancements, with new agents being developed to target specific symptoms and underlying mechanisms. Current pharmacological options include antispasmodics, antidepressants, and emerging therapies specifically designed for IBS, such as eluxadoline and rifaximin. In particular, these emerging therapies can relieve IBS-D [69, 70]. A multi-center study by Di Nardo et al. [71] demonstrated the safety and efficacy of jointly micronized palmitoylethanolamide/polydatin for treating abdominal pain in pediatric patients with IBS, especially those with the IBS-D subtype. Another clinical trial illustrated that ebastine, a pre-existing drug, may serve as a novel treatment for IBS that is not characterized by constipation [69]. In addition, tenapanor has been identified as a new therapeutic option for IBS-C [70]. Similarly, plecanatide treatment improved abdominal and bowel symptoms in IBS-C [72].

**Table 2:** Summary of clinical trials for IBS treatment (Western and traditional Chinese medicine)

Reference number	Research type	IBS subtype	Sample size	Intervention	Study results
[69]	Multicenter, randomized, double-blind, placebo-controlled trial	Non-constipated IBS	202	Ebastine	Ebastine improved global symptoms and abdominal pain
[71]	Multicenter randomized, double-blind, placebo-controlled, parallel-arm trial	IBS-C IBS-D IBS-M	70	Palmitoylethanolamide/polydatin	Palmitoylethanolamide/polydatin relieved abdominal pain
[72]	Randomized, double-blind, placebo-controlled trials	IBS-C	1436	Plecanatide	Plecanatide improved IBS-C abdominal and bowel symptoms
[74]	Randomized, double-blind, placebo-controlled, parallel-group, multicenter study	IBS-D	200	*Bifidobacterium longum* CECT 7347, heat-treated *Bifidobacterium longum* CECT 7347	*Bifidobacterium longum* CECT 7347 and heat-treated *Bifidobacterium longum* CECT 7347 reduced IBS-D symptoms
[75]	Randomized, double-blind, and placebo-controlled trial	IBS-C	30	*Lacticaseibacillus rhamnosus* IDCC 3201	*Lacticaseibacillus rhamnosus* IDCC 3201 ameliorated IBS-C
[78]	Single-center prospective study	IBS-C IBS-D	17	FMT	FMT improved the IBS severity index
[79]	Randomized, double-blind, placebo-controlled study	IBS-C IBS-D IBS-M	165	FMT	FMT improved IBS symptoms
[81]	Single-center, double-blind, randomized, placebo-controlled trial	IBS-D	56	FMT	FMT improved IBS-induced abdominal bloating
[82]	Randomized, Placebo-controlled trial	Not specified	49	FMT	FMT could not relieve IBS symptoms
[86]	Multicenter, randomized, sham-controlled trial	IBS-C IBS-D IBS-M	170	Acupuncture	Acupuncture alleviated refractory IBS symptoms

FMT: Fecal microbiota transplantation; IBS: irritable bowel syndrome; IBS-C: constipation-predominant IBS; IBS-D: IBS-Diarrhea; IBS-M: mixed-diarrhea-and-constipation IBS

The role of probiotics in IBS management is linked to gut microbiota modulation [73]. Clinical evidence shows that they significantly alleviate abdominal pain and bloating in patients with IBS, with notable efficacy in IBS-D [73–76]. Specifically, *Bifidobacterium longum* CECT 7347 (including its heat-processed postbiotic form) effectively reduced IBS-D symptoms [74], whereas *Lacticaseibacillus rhamnosus* IDCC 3201 ameliorated IBS-C via gut microbiota enrichment and metabolite regulation [75]. However, probiotic efficacy is strain-specific and varies. The low-fermentable oligosaccharide, disaccharide, monosaccharide, and polyol diet (low-FODMAP diet) enhances probiotic effectiveness by reducing fermentable substrates [68]. In addition, this diet can rapidly relieve bloating and defecation abnormalities independently. However, long-term adherence is challenging, potentially decreasing gut microbiota diversity.

Dietary intervention exhibits promising clinical potential in regulating the microbiota–mitochondria axis. The combination of low-FODMAP diets and probiotics (e.g., *B. longum* CECT 7347 and *L. rhamnosus* IDCC 3201) synergistically enhances IBS treatment efficacy. Low-FODMAP diets alleviate mitochondrial stress by reducing fermentable substrate accumulation, whereas probiotics optimize gut microbiota structure and promote the production of beneficial metabolites that support mitochondrial function [68, 74, 75].

FMT is an intervention aimed at reestablishing microbial balance, with some patients reporting symptom relief [73, 77–79]. However, most existing trials are single-center studies with small sample sizes (mostly ≤ 100 cases), inconsistent administration routes (endoscopic vs. capsule delivery), and short follow-up periods (mostly < 12 months)—methodological limitations that contribute to significant efficacy heterogeneity. A meta-analysis of seven RCTs suggested potential symptom improvement [80]. Nonetheless, critical appraisal reveals notable inconsistencies across studies. For instance, Yau et al. [81] observed improvement only in abdominal bloating without reduction in IBS-SSS scores, indicating limited efficacy for core symptoms (abdominal pain and altered bowel habits) and warranting caution in clinical application. Additionally, conflicting evidence has emerged from a study using the 16 S rRNA gene amplicon and shotgun metagenomics, as well as a separate meta-analysis [82, 83], further undermining the robustness of current findings. Given that FMT effectiveness is highly influenced by donor screening criteria and administration protocols, future research should prioritize multi-center, large-sample RCTs that standardize these variables to fully evaluate its true therapeutic value [73, 77–83].

Although various Western treatments have been proposed, large-scale clinical RCTs controlling confounders such as location and diet must be carefully conducted to determine the most effective treatment for IBS. Recent updates in clinical guidelines have emphasized the need for individualized treatment that considers the diverse clinical manifestations of IBS and varying responses to different treatments [67, 84].

### TCM treatment progress of IBS

TCM has been integrated into the management of IBS. It offers alternative therapeutic strategies centered on restoring bodily balance, including herbal formulations, acupuncture, and dietary recommendations, which can relieve symptoms and improve intestinal health. Recent research has underscored the clinical efficacy of specific TCM interventions in the management of IBS symptoms, particularly in improving gut motility and reducing inflammation [68, 85]. Acupuncture can enhance gut motility and reduce visceral hypersensitivity in patients with refractory IBS (Table 2) [86]. Similarly, the benefits of moxibustion have been confirmed for IBS [87]. Herbal compounds such as Baizhu Shaoyao Decoction [88], Tongxie Yaofang Decoction [89], Sishen Wan [90], and Da-Jian-Zhong Decoction [91] have demonstrated therapeutic effects on IBS-D in animal models. Meta-analyses and reviews have supported the efficacy of Chinese herbal medicines in treating IBS [92, 93]. A growing body of research confirms the applicability of TCM in IBS treatment, indicating that it can complement conventional therapies and provide a more comprehensive management plan for patients [94]. However, TCM interventions have some limitations. First, existing research has not fully elucidated their mechanisms of action [68] Moreover, not all the aforementioned therapeutic strategies have been sufficiently validated by clinical studies, with some lacking evidence-based support [85]. In addition, the lack of unified efficacy evaluation criteria and the complex composition of some herbal formulations that may hinder the clinical application of TCM for IBS treatment. Therefore, more rigorous clinical trials based on existing basic research are required. The limitations of existing treatments have prompted researchers to explore innovative therapies targeting the mitochondria–gut microbiota axis.

### IBS treatment targeting mitochondria and the gut microbiota: Novel findings

Clinical trials of novel therapeutic approaches for IBS have produced promising results. These studies have demonstrated the efficacy of interventions targeting gut microbiota modification and mitochondrial function enhancement. A notable advancement in this direction has been the exploration of dietary interventions, especially low-FODMAP diets. A systematic review showed that adherence to this diet significantly alleviated abdominal pain and bloating and enhanced the global quality of life in patients with IBS. Many patients reported sustained symptom relief even after reintroduction of the diet [95].

In addition to dietary modifications, clinical trials have evaluated the use of probiotics as a therapeutic strategy for IBS. As a key intervention for regulating the gut microbiota, probiotics primarily supplement beneficial strains to improve the structure of the microbiota, thereby alleviating IBS symptoms. A meta-analysis of several RCTs revealed that specific probiotic strains can remarkably reduce IBS symptoms, particularly in patients with IBS-D [96]. However, the variability in responses is largely attributed to unaddressed methodological flaws in individual trials, including a lack of strain-specific mechanism exploration, inadequate baseline stratification, and placebo effect interference. This highlights the need for personalized treatment approaches based on microbiota typing, as not all individuals respond uniformly to probiotic supplementation. Future trials must address these limitations to validate the efficacy of targeted probiotic therapies.

FMT has also emerged as a novel therapeutic strategy for IBS, particularly in cases in which traditional treatments fail. Recent investigations have emphasized its efficacy in the restoration of gut microbiota diversity and functionality—features frequently perturbed in patients with IBS [97]. Some clinical trials have reported that FMT may lead to symptom relief in certain patients with IBS [98, 99], However, critical appraisal reveals obvious individual variation in efficacy rooted in methodological limitations. As highlighted earlier, most trials have small sample sizes, non-standardized donor screening, and short follow-ups, leading to inconsistent results. Thus, the long-term safety and efficacy of this intervention remain to be confirmed by more high-quality studies [99]. In particular, future trials should prioritize addressing these limitations to clarify the clinical value of FMT in IBS.

Some preliminary studies suggest that FMT indirectly affects mitochondrial function by regulating the gut microbiota [100], and that restoration of a healthy microbiome could theoretically enhance mitochondrial bioenergetics. If confirmed, this association may address two interrelated aspects of IBS pathogenesis. However, the link itself and its clinical significance have not been clearly elucidated and require further verification. Animal models, particularly rodent models, are crucial for clarifying how mitochondrial dysfunction and gut microbiota dysbiosis contribute to IBS [101]. For example, stressed mice exhibited impaired mitochondrial function and altered gut microbiota similar to those reported in patients with IBS [102]. Additionally, mitochondrial dysfunction increases oxidative stress, worsens gut inflammation and dysbiosis, and promotes IBS [25]. Interventions that restore mitochondrial function have shown improvement in IBS-like symptoms in animal models [103]. However, this potential requires further verification for clinical application, as translation from preclinical data to human efficacy remains unconfirmed. Current mitochondria-targeted therapies for IBS are mostly preclinical studies or small-sample preliminary explorations. Preliminary results suggest that IBS symptoms are alleviated by enhancing mitochondrial bioenergetics and reducing gut oxidative stress [104]. However, their clinical translational value needs verification through large-sample, long-term follow-up clinical trials. With a deeper understanding of the gut–mitochondria link, these therapies could provide new avenues for IBS treatment.

In summary, recent clinical trials on new therapeutic strategies for IBS have underscored the significance of the gut microbiota and mitochondrial function in the pathophysiology of this disease. Dietary changes, probiotics, FMT, and mitochondria-targeted therapies are promising treatment strategies. Further clinical and basic research is required to optimize these approaches, account for individual treatment differences, and improve the quality of life of patients with IBS.

### Future research direction on the treatment of IBS

#### Potential therapies for improving mitochondrial function

The exploration of mitochondria-targeted therapies for IBS is still at an early stage, with several innovative approaches under preliminary investigation. These include the use of mitochondrial antioxidants and compounds that enhance mitochondrial biogenesis to counteract oxidative stress and energy deficits in patients with IBS [3]. Recent breakthroughs in nanomedicine have also shown the potential to deliver therapeutic agents directly to mitochondria, thereby improving their efficacy and reducing systemic side effects [105]. Moreover, targeting the gut microbiota through mitochondrial modulation has been proposed as a dual strategy to restore mitochondrial function and microbiome balance in cancers, which may improve clinical outcomes in patients with IBS [106]. With continuous advancements in clinical research, the integration of such therapies into IBS treatment paradigms remains hypothetical and requires robust clinical evidence to support its feasibility.

Mitochondrial dysfunction is progressively acknowledged as a vital factor in numerous diseases, including neurodegenerative disorders, cardiovascular diseases, and metabolic syndromes. Potential intervention directions for improving mitochondrial function include the application of mitochondrial antioxidants and compounds that promote mitochondrial biogenesis, nano-drug targeted delivery technology, and dual strategies combining gut microbiota regulation. Furthermore, lifestyle interventions such as exercise can enhance mitochondrial density and function, providing auxiliary support for IBS treatment. For instance, mitochondrial replacement therapies and mitochondria-targeted antioxidants have shown promise in preclinical studies, particularly against conditions in which mitochondrial impairment holds considerable significance in the pathophysiology, including Huntington’s disease and spinal cord injury [107, 108]. Additionally, exercise has been identified as a natural method for improving mitochondrial function, particularly in skeletal muscles, where it enhances mitochondrial density and biogenesis to ameliorate conditions such as chronic obstructive pulmonary disease [109]. However, future research should clarify the precise mechanisms through which these therapies elicit their effects, optimize dosages, and identify patient populations that would benefit the most from such interventions.

Mitochondria-targeted therapies represent a burgeoning area of research with the potential to revolutionize treatment paradigms for various diseases. These therapies restore mitochondrial function, improve bioenergetics, and reduce oxidative stress. For example, recent research has explored the application of mitochondria-targeted antioxidants (e.g., MitoQ) in conditions such as COVID-19 and neurodegenerative diseases [110, 111]. Innovative approaches such as mitochondrial transplantation have shown preclinical potential in relevant disease models [112]. However, insufficient clinical evidence currently supports their application in IBS, and their safety and efficacy need to be verified by studies specifically targeting patients with IBS. Future investigations should prioritize the combined validation of mitochondria-related biomarkers (e.g., mitochondrial DNA copy number and ATP 6/8 gene expression) and microbial metabolites (e.g., SCFAs and bile acids), while emphasizing large-sample, placebo-controlled RCTs to validate the efficacy and safety of mitochondria-targeted therapies. This combined validation requires rigorous cohort studies and diagnostic trials to clarify the sensitivity, specificity, and predictive value of these biomarkers in IBS diagnosis, subtype differentiation, and efficacy assessment. For clinical trials on mitochondria-targeted therapies, efforts should be made to explore optimal dose gradients, identify suitable patient populations based on mitochondrial function profiles, and adopt standardized outcome measures to ensure the reliable comparability of results across different studies. The integration of nanotechnology to enhance the delivery efficiency of mitochondria-targeted therapies has been investigated in fields such as cancer therapy and chronic disease management [113]. However, its applicability and value in IBS treatment remain unproven and require further exploratory research.

#### Clinical research on gut microbiota interventions

The gut microbiota plays a pivotal role in both disease and health by influencing metabolic, immunological, and neurological functions. Recent clinical investigations have explored the therapeutic potential of gut microbiota-targeted interventions across diverse conditions, including obesity, diabetes, and IBD. For instance, interventions such as probiotics, prebiotics, and FMT have shown potential in regulating gut microbiota composition in patients with IBD [114]. However, these results cannot be directly generalized to IBS. The efficacy of FMT in this disorder requires large-sample RCTs specifically designed for IBS populations. These trials should standardize donor screening criteria (e.g., microbial diversity and absence of pathogenic bacteria), optimize administration frequency and routes, and establish a unified efficacy evaluation system to address heterogeneity. Subgroup analyses based on patient subtypes and baseline microbiome profiles are also required to identify responders. Additionally, the association between exercise and gut microbiota alterations has been documented, suggesting that lifestyle interventions can exert a positive effect on gut health [115]. Future clinical studies, especially RCTs, should be conducted to evaluate the therapeutic effects of microbiota intervention such as FMT, optimize treatment protocols, and identify specific patient populations that may benefit from microbiota-targeted therapies. In addition, basic research clarifying the mechanisms underlying these interventions and exploring relevant biomarkers to evaluate the therapeutic effects should be conducted.

#### Exploration of personalized and optimized comprehensive treatment

The movement toward personalized medicine has garnered increasing interest, particularly within the framework of mitochondrial dysfunction and gut microbiota intervention. Comprehensive treatment strategies that incorporate individual patient characteristics, such as microbiome profiles, genetic predispositions, and lifestyle factors, can markedly maximize therapeutic outcomes. For example, integrating mitochondria-targeted therapies with lifestyle modifications, including dietary alterations and physical exercise, may synergistically enhance mitochondrial function and overall health [116]. In addition, personalized approaches to gut microbiota interventions tailored to individual microbiome profiles could enhance treatment efficacy in conditions such as diabetes and obesity [117]. Thus, future studies should emphasize the development of frameworks for personalized treatment plans while incorporating advanced approaches such as genomics and metabolomics to guide therapeutic decisions.

### Challenges and opportunities for future research

Several challenges and opportunities have emerged as the fields of mitochondrial science and gut microbiota research have evolved. One critical challenge is the complexity of mitochondrial dysfunction and its multifactorial nature, which hinders the development of effective therapies. More basic scientific research using multi-omics data, personalized microbiome mapping, and mitochondrial functional assays that focus on defining clear mechanistic links between mitochondrial metabolism and microbial signaling in IBS is required in future studies. Moreover, ethical considerations regarding personalized medicine, particularly data privacy and patient consent, must be addressed with advancement in scientific research. However, these challenges present opportunities for interdisciplinary collaboration, innovative research designs, and potential groundbreaking discoveries that could transform therapeutic approaches for various diseases. Future research should investigate the mitigation of these obstacles while leveraging advancements in technology and scientific understanding to enhance the quality of patient care and therapeutic efficacy. In addition, the integration of emerging technologies, such as machine learning and artificial intelligence, into research methodologies presents both challenges and opportunities for data analysis and interpretation [118].

## Discussion

The interrelationship between mitochondrial function and the gut microbiota represents a critical research focus for elucidating the pathophysiology of IBS. Current research underscores the significance of these two biological systems. Mitochondrial dysfunction can exacerbate gut dysbiosis, which may contribute to IBS symptoms via neuroendocrine and immune pathways. Nevertheless, it is imperative to acknowledge the limitations of existing research, including small sample sizes, varied methodologies, and a lack of longitudinal data, and the fact that most studies are merely correlational. Therefore, further longitudinal cohort studies are required to verify the temporal sequence and causal direction of the mitochondria–gut microbiota interaction, as these factors hinder the establishment of definitive causal relationships and the generalizability of study findings.

Future basic research should adopt a multi-center, large-sample longitudinal cohort designs, integrating multi-omics technologies (genomics, metabolomics, and microbiomics) to elucidate the dynamic interaction mechanisms of the mitochondria–gut microbiota axis in IBS. This will help clarify the specific molecular signatures of different IBS subtypes (IBS-C/IBS-D) and delineate key signaling pathways underlying their crosstalk. Targeting these pathways could pave the way for innovative treatments that address not only the symptoms of IBS but also the underlying dysregulation of the gut microbiota and mitochondrial systems. Long-term studies monitoring variations in mitochondrial activity and microbiome composition over time may offer valuable insights into the temporal dynamics of IBS. Additionally, exploring the specific mitochondrial pathways and metabolic profiles associated with different IBS phenotypes could facilitate the development of individualized therapeutic strategies. Meanwhile, sex- and gender-related differences may jointly participate in the pathogenesis of IBS by affecting gut microbiota composition (e.g., enrichment of harmful bacteria in women) and mitochondrial function (e.g., abnormal expression of energy metabolism-related genes in men), consequently providing directions for future sex-targeted research.

A holistic view is required in the study of IBS pathophysiology. Integrating microbiology, mitochondrial biology, immunology, and metabolic research can help better understand IBS, identify biomarkers, and develop more effective therapies. In addition, clinicians should integrate basic research findings into clinical practice and conduct rigorous clinical trials. Future trials should adopt standardized efficacy evaluation systems (e.g., unified IBS-SSS scoring criteria and intestinal barrier function detection indicators) to reduce research heterogeneity. Furthermore, future trials should implement long-term follow-ups (≥ 12 months) to verify the stability and safety of intervention effects. Patient-centric approaches are also important when considering patient diversity in personalized treatment. Although interventions targeting mitochondrial function and the gut microbiota offer an exploratory direction for IBS treatment, the current evidence base remains preliminary. While advances in microbiome research and mitochondrial biology may yield new insights, innovative therapeutic strategies require validation through additional high-quality clinical studies. Existing research is limited by small sample sizes, short follow-up durations, and heterogeneous study designs—factors that have precluded definitive confirmation of efficacy stability and optimal patient populations. Complementary approaches, such as probiotics, prebiotics, and lifestyle modifications aimed at enhancing mitochondrial efficiency, may hold promise for alleviating IBS symptoms. However, their clinical utility similarly requires further investigation.

In conclusion, embracing a multidisciplinary perspective that considers the contributions of mitochondrial health and the gut microbiota will remain pivotal as we unravel the complexities of IBS. Ultimately, future in-depth studies adopting integrated methodologies, including multi-omics-based longitudinal cohorts, standardized clinical trials, and combined biomarker validation, will clarify the complex interaction between mitochondria and the gut microbiota. This will facilitate the development of personalized, targeted strategies, thereby transforming the landscape of IBS treatment and enhancing patient quality of life.

## Data Availability

No data were used or generated for the research described in the article.

## Abbreviations

IBS: Irritable Bowel Syndrome
SCFAs: Short-chain Fatty Acids
ROS: Reactive Oxygen Species
ATP: Adenosine Triphosphate
FMT: Fecal Microbiota Transplantation
IL-1β: Interleukin-1β
IBS-D: IBS-Diarrhea
IBS-M: Mixed-Diarrhea-and-Constipation IBS
16S rRNA: 16 S Ribosomal Ribonucleic Acid
TNF-α: Tumor Necrosis Factor-α
IL-6: Interleukin-6
NLRP3: NOD-like Receptor Pyrin Domain-Containing Protein 3
IBD: Inflammatory Bowel Disease
IBS-C: Constipation-Predominant IBS
low-FODMAP diets: Low-Fermentable Oligosaccharides, Disaccharides, Monosaccharides and Polyols Diets
TCM: Traditional Chinese Medicine
COVID-19: Coronavirus Disease 2019
